# High-Quality Ferromagnetic Josephson Junctions Based on Aluminum Electrodes

**DOI:** 10.3390/nano12234155

**Published:** 2022-11-24

**Authors:** Antonio Vettoliere, Roberta Satariano, Raffaella Ferraiuolo, Luigi Di Palma, Halima Giovanna Ahmad, Giovanni Ausanio, Giovanni Piero Pepe, Francesco Tafuri, Davide Massarotti, Domenico Montemurro, Carmine Granata, Loredana Parlato

**Affiliations:** 1 Consiglio Nazionale delle Ricerche—ISASI, Via Campi Flegrei 34, I-80078 Pozzuoli, Italy; 2 Dipartimento di Fisica “Ettore Pancini”, Università degli Studi di Napoli Federico II, I-80125 Napoli, Italy; 3 Consiglio Nazionale delle Ricerche—SPIN, c/o Complesso Monte Sant’Angelo, via Cinthia, I-80126 Napoli, Italy; 4 Dipartimento di Ingegneria Elettrica e delle Tecnologie dell’Informazione, Università degli Studi di Napoli Federico II, I-80125 Napoli, Italy; 5 Consiglio Nazionale delle Ricerche—Istituto Nazionale di Ottica (CNR-INO), Largo Enrico Fermi 6, I-50125 Florence, Italy

**Keywords:** aluminum Josephson junctions, hybrid ferromagnetic Josephson junction, quantum computing applications

## Abstract

Aluminum Josephson junctions are the building blocks for the realization of superconducting quantum bits. Attention has been also paid to hybrid ferromagnetic Josephson junctions, which allow switching between different magnetic states, making them interesting for applications such as cryogenic memories, single-photon detectors, and spintronics. In this paper, we report on the fabrication and characterization of high-quality ferromagnetic Josephson junctions based on aluminum technology. We employed an innovative fabrication process inspired by niobium-based technology, allowing us to obtain very high-quality hybrid aluminum Josephson junctions; thus, supporting the use of ferromagnetic Josephson junctions in advanced quantum circuits. The fabrication process is described in detail and the main DC transport properties at low temperatures (current–voltage characteristic, critical current as a function of the temperature, and the external magnetic field) are reported. Here, we illustrate in detail the fabrication process, as well as the main DC transport properties at low temperatures (current–voltage characteristic, critical current as a function of the temperature, and the external magnetic field).

## 1. Introduction

Research on low-temperature Josephson junctions (JJs) has been boosted by application demands [1] for superconducting digital circuits [2], highly sensitive magnetometers [3], and radiation detectors [4]. JJs have attracted great interest as building blocks of superconducting quantum circuits, enabling the observation of quantum phenomena at the macroscopic level and are commonly used in quantum processors [5]. One of the main benefits of the superconducting quantum bit (qubit) is that its fabrication is fully compatible with the well-established semiconducting technology, and it allows wide design flexibility and scalability [6]. In the last two decades, the coherence times of superconducting qubits have increased by more than five orders of magnitude, mainly through improvements in the design, fabrication, and the choice of their component materials and interfaces [7]. Among JJs fabricated with different superconducting materials, Al/AlO_x_/Al junctions are the most widely used due to the large coherence time they guarantee [8,9].

For the integration in quantum circuits, JJs are designed to have large sub-gap resistance to reduce quasi-particle noise [10,11], and an appropriate *E_J_*/*E_C_* ratio, where *E_J_ = (ħI_c_)*/2*e* is the Josephson energy with *I_c_* as the critical current, and *E_C_ = e*^2^/2*C* is the charging energy associated to the junction capacitance *C*. In superconducting quantum architectures [12,13,14], miniaturization becomes an important issue not only for the integration of multiple qubits on the same chip, but also to achieve suitable values of *E_J_* in single qubits by reducing the lateral size of the JJ. The most common and robust process for fabricating nanoscale JJs consists of the fabrication of Al/AlO_x_/Al sandwiches by electron-beam lithography and double-angle shadow evaporation of aluminum in a single vacuum cycle [15,16].

In this paper, we report on the fabrication of high-quality micron-sized aluminum (Al) JJs based on a different approach. The fabrication uses the notions and the recipe optimized for Nb tunnel junctions, which is based on the anodization of the Al top layer and an additional silicon dioxide insulating layer [17,18]. This provides a reliable and reproducible process to obtain high-quality JJs with Al electrodes down to 12 μm^2^ through standard optical lithography with *E_J_* values suitable for integration in a transmon quantum architecture.

Furthermore, our process allows the deposition of additional layers, and specifically of the ferromagnetic (F) layer afterward the definition of the junction. The overall structure is a magnetic JJ (MJJ) of the type SIS’FS; i.e., a superconductor/insulator/thin superconductor/ferromagnet/superconductor stacked multilayer [19]. So far, tunnel SIS’FS JJs have been designed using standard Nb technology for the implementation of cryogenic superconducting magnetic memories compatible with single-flux-quantum (SFQ) circuitry. The fabrication process is flexible and allows the use of different ferromagnetic materials, either soft (PdFe [20,21]) or strong (Py [22]). The magnetic nature of the SIS’FS junction guaranteed by the F layer is confirmed by the hysteretic behavior of the magnetic field pattern, while preserving the high quality of the tunnel behavior guaranteed by the Al oxide barrier. This technology can be extended to most ferromagnetic materials to develop ad hoc switchable elements, and guarantee the easy integration of MJJs in a large variety of digital and quantum circuits by standard optical lithography [19]. In addition, the developed fabrication approach is reproducible and adaptable to a wide class of fabrication protocols since ferromagnetic materials can be deposited ex situ without affecting the tunnel properties of the overall device. Recently, it has been proposed that the memory properties of MJJs can provide an alternative tuning of the qubit frequency upon the application of magnetic field pulses in a hybrid superconducting qubit, the so-called ferro-transmon [23]. In this context, our results are a fundamental step toward the integration of Al-based MJJs in hybrid quantum architectures. Hybrid SIS’FS junctions can be also exploited as microwave single-photon detectors since they provide optimal control and tunability of the critical current [24]. Finally, in the field of cold-electron bolometers, Al/Fe structures with tunnel barriers allow the fabrication of thinner absorbers; thus, improving their sensitivity [25]. 

## 2. Fabrication Process

**Figure 1:**
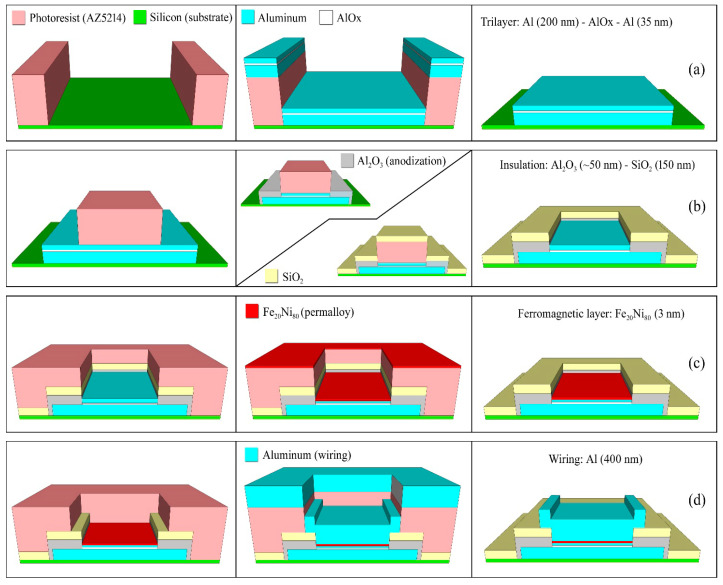
Sketch of the fabrication process: (**a**) definition, deposition, and shaping of the trilayer (Al–AlOx–Al) by lift-off procedure; (**b**) definition of JJ area, and insulation by selective anodization process and silicon dioxide deposition; (**c**) definition, deposition, and lift-off of ferromagnetic layer; and (**d**) wiring.

The device fabrication starts from a trilayer deposition in an ultra-high vacuum system down to 3 × 10^−8^ Torr. A multilayer structure consisting of Al/AlO_x_/Al is deposited on a patterned silicon wafer by means of a magnetron DC-sputtering procedure. The JJ geometry is obtained by a standard optical photolithography and lift-off process. The vacuum chamber is equipped with two-inch magnetron sources powered by a DC or RF generator with a maximum power of 500 W. One of the magnetrons has an aluminum target (purity 99.9%). Inside the chamber, three-inch silicon wafers are placed on a rotating platform with six available positions. Between the magnetrons and the rotating platform, there is a fixed stage that allows placing only one sample at a time facing the source. The pumping system consists of a turbomolecular group with an ion pump and a titanium sublimator that allow reaching a base vacuum of about 10^−9^ torr. Before each deposition, a pre-sputtering operation is carried out far from the wafer device, in order to clean the target surface from impurities accumulated during the loading and unloading operations. The base 200 nm thick Al layer was deposited by dc sputtering at a rate of 0.8 nm/s by using an argon (Ar) process gas at a pressure of 3.5 mTorr. The working voltage and current of the magnetron are 400 V and 0.28 A, respectively. To avoid heating problems, the deposition is carried out in two identical steps with a waiting time of 2 h. The tunnel barrier is obtained by exposing the chamber to dry oxygen at a pressure of 200 mTorr for 1 h. Afterward, the Al top layer of 35 nm thickness is deposited in the same conditions as for the base layer. The lift-off procedure consists in dissolving the photoresist in acetone at room temperature for 2 h (Figure 1a).

The Josephson junctions, having a square shape with an area ranging from 12 to 80 µm^2^, are patterned again by optical photolithography. Through an anodization process, described below, we completely oxidize the top aluminum in the area surrounding the region covered by the photoresist; thus, defining the area of the junctions (Figure 1b). Then, the sample is immersed in an electrolyte solution consisting of 1 part of deionized water, 1.5 parts of glycol ethylene, and 20 g of ammonium pentaborate per 100 mL of deionized water. The cathode is made of a platinum electrode, while the Al top layer represents the anode. The circuit is powered by a constant current. As the current flows, the voltage increases because of the formation of Al_2_O_3_, observed at the ends of the electrodes. An increase of 1 V corresponds to the anodization of about 1 nm of Al and an increase in the thickness of the oxide of about 1.5 nm. The process is monitored by plotting the time derivative of the voltage across the cell versus the voltage itself. The rate at which aluminum oxide grows is a crucial parameter. If it is fast, the oxide could consist of large grains and does not ensure a good insulator; on the other hand, if it is quite slow, it could affect the small junctions (with an area lower than 100 μm^2^) due to the undesired anodization even under the edges of the photoresist. A good compromise is therefore required. The growth rate of the oxide is proportional to the current with which the anodizing cell is biased. We set the anodization speed at 0.4 V/s, corresponding to an oxide growth rate of about 0.6 nm/s. The process is stopped when the value of 40 V is reached at the ends of the cell, which assures that all the top aluminum layer is anodized. After the anodization process, the samples are inserted in another high-vacuum system to deposit an additional insulation layer (Figure 1b). A thin silicon dioxide layer having a thickness of 150 nm is deposited by using a 2-inch magnetron powered with a RF generator at a rate of 0.5 nm/s. The Ar gas process is at a pressure of 3.0 mTorr, while the generator power is 150 W. This additional oxide layer guarantees better insulation; and therefore, reduces the sub-gap leakage currents, improving the quality of the JJs. It is worth noting that for superconducting qubit applications, we will consider to replace the SiO_2_ with another insulator to avoid dielectric losses and two-level defects, which may affect the qubit coherence [7,26].

**Figure 2:**
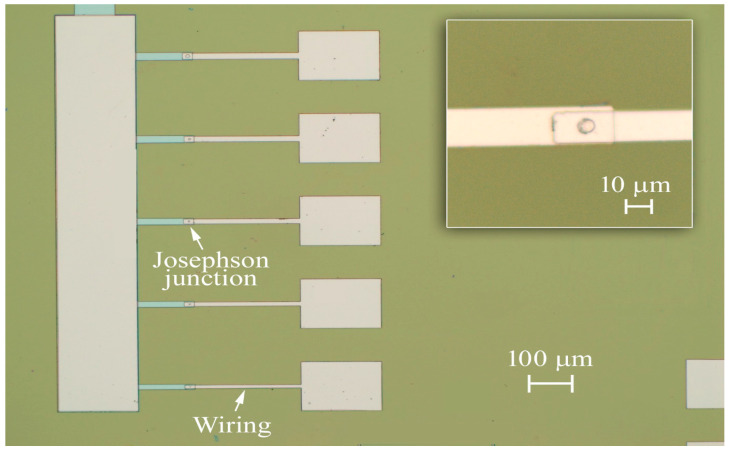
Optical microscope image of a set of circular SIS’FS having a diameter ranging from 2 to 10 μm. In the inset, the magnification of a circular junction with a diameter of 4 μm is displayed.

Then, a soft Ar ion cleaning of the Al surface was performed before the deposition of the F layer (permalloy: Py) by DC magnetron sputtering (Figure 1c). In the third step, after a soft surface cleaning conducted by the Argon ion gun, the F layer (permalloy) was deposited by DC sputtering (Figure 1c). The thickness of the Py layer, having a stoichiometry of 83 (± 3) % Ni and 17 (± 3) % Fe, is 3 nm to avoid the suppression of the superconducting current induced by the S/F proximity effect [22]. Finally, after the last photolithography step to define the geometry of the device wiring, a thicker layer (400 nm) of aluminum film is deposited in the same condition and at the same rate as the base and top aluminum described above. Again, to avoid heating problems, the wafers are thermally anchored to a copper support to dissipate the heat and the deposition is carried out in two steps with a waiting time of 2 h. The anchoring is done by using Apiezon^®^ L vacuum grease exhibiting an extremely low vapor pressure of 7 × 10^−11^ Torr at 20 °C. In the case of Al/AlO_x_/Al JJs, a low-power ion gun cleaning is performed before the wiring deposition to remove the surface oxide. The process is completed by a lift-off procedure by placing the samples in acetone at room temperature for about 3 h (Figure 1d). In Figure 2, we report an optical image of a junction set and a circular junction having an area of about 12 μm^2^ in the inset. 

## 3. Results and Discussion

The devices were anchored to the mixing chamber of a dry dilution refrigerator Oxford Triton 400, equipped with customized RC and copper-powder low-pass filters, to be characterized down to 10 mK. More details on the measurement setup are reported elsewhere [27,28,29]. The current–voltage (I–V) curves are measured as a function of the temperature and magnetic field by current biasing the samples and by measuring the voltage across the junction. The voltage signal is amplified by differential amplifiers, while the magnetic field is generated by a superconducting NbTi coil. 

For the differential conductance data, the input current is the sum of two signals: a small sinusoidal excitation of about 30 Hz superimposed on a triangular ramp with a frequency of about 1 mHz. A magnetic field that corresponds to one of the minima of the *I_c_*(*H*) pattern is applied to suppress the Josephson current of the junction.

**Figure 3:**
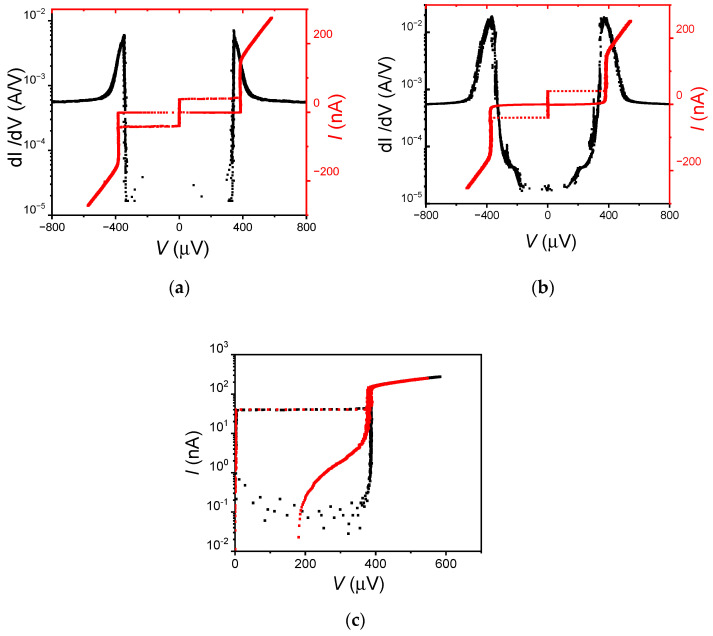
(**a**) Conductance *dI/dV* measurement and I–V curve for the SIS’S junction with a diameter of 4 μm; (**b**) conductance measurement and I–V curve for the SIS’FS junction with a diameter of 4 μm. The conductance measurements were performed by applying a magnetic field of 10 mT to suppress the Josephson supercurrent. (**c**) Magnification of the positive part branch of the I–V curve in a log-scale for both the SIS’S (black curve) and the SIS’FS (red curve) junction with a diameter of 4 μm.

Typical I–V curves for SIS’S and SIS’FS junctions at T = 10 mK are reported in Figure 3. The critical current density *J_c_* is about 0.4 A/cm^2^, which is almost independent of the junction area. Both SIS’S and SIS’FS JJs show the high quality of the tunnel barrier, which is evident from the shape of the sub-gap branch. This behavior can be described in the frame of the tunnel junction microscopic model (TJM) [30,31]. By fitting the I–V characteristics in Figure 3 with the TJM model, we estimated the sub-gap resistance *R_sg_* of the order of a few MΩ for both the SIS’S and SIS’FS JJs; and hence, a ratio *R_N_/R_sg_* of about 10^−3^ with *R_N_*, the normal resistance of the JJ. This estimation is in agreement with the very low values of the measured leakage currents lower than 0.5 nA, which is the resolution limit of our experimental setup. Moreover, the characterization of different fabrication batches shows a similar performance, guaranteeing good repeatability of the fabrication process.

In the conductance measurements in Figure 3b, we observe the appearance of two symmetric structures at *V* ≈ 200 μV, i.e., at a voltage $V\;\sim V_{gap}/2$ with *V_gap_*, the voltage gap of the junction. These structures are not specific features of magnetic JJs; however, they can be found in standard tunnel JJs [32], where they are typically ascribed to a coherent tunneling mechanism of multiple quasiparticles [33,34]. The conductance behavior and the low values of the sub-gap currents further confirm the high quality of the junctions, which are not affected by the presence of the F layer.

The characteristic voltage *V_c_ = I_c_R_N_* is about 25% of the expected Ambegaokar-Baratoff value [35]. The suppression of *I_c_* could be related to the presence of paramagnetic impurities [36] or oxygen vacancies in the insulating barrier [37]. The suppression of *I_c_* was observed in JJs employed in quantum circuits and does not seem to affect their coherence times [38].

**Figure 4:**
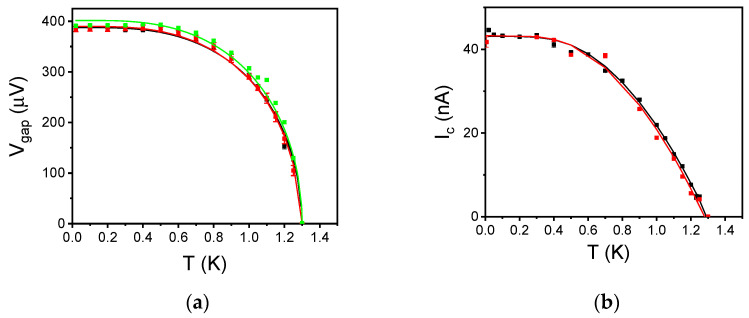
(**a**) Behaviour of *V_gap_* as a function of the temperature for a circular SIS’S junction with a diameter of 4 μm (dark curve), and SIS’FS junctions with a diameter of 4 μm (red curve) and 10 μm (green curve), respectively. The solid curves are the fitting curves calculated by using Equation (1). (**b**) Behaviour of critical current *I_c_* as a function of the temperature for a circular SIS’S junction with a diameter of 4 μm (dark curve) and SIS’FS junctions with a diameter of 4 μm (red curve). The solid curves are the fitting curves calculated by using Equation (2).

In Table 1, the main parameters of the measured junctions are reported. The behavior of the *V_gap_* (*V_gap_ =* 2Δ/*e*) and the critical current *I_c_* as a function of the temperature up to the Al critical temperature (*T_c_* ≈ 1.3 K) are shown in Figure 4. The experimental *V_gap_*(*T*) and *I_c_*(*T*) curves agree with the Bardeen–Cooper–Schrieffer (BCS) approximation in the weak-coupling limit [30] and the Ambegaokar-Baratoff relation [35]:

(1) $$V_{gap}\left(T\right)=\frac{2\Delta_{0}}{e}tanh\left(1.74\sqrt{1-\frac{T}{T_{C}}}\right)\;$$

(2) $$I_{C}R_{N}=B\frac{\pi }{2}\frac{\Delta \left(T\right)}{e}tanh\left(\frac{\Delta \left(T\right)}{2k_{B}T}\right),$$

Respectively; here, *B* is a fitting coefficient that takes into account the suppression with respect to the theoretical value. Note that the difference in the critical current density between the 4 μm and 10 μm SIS’FS is probably due to the anodization process, which also occurs under the photoresist pattern, leading to a JJ effective area less than the geometrical one. This effect has a major impact on the smallest junctions. From the fitting parameters reported in Table 1, we conclude that the developed multi-step fabrication procedure allows building magnetic tunnel JJs preserving all the features of Al tunnel JJs. In addition, since the ex situ deposition of the F-layer avoids any magnetic contamination of the UHV system where the deposition of the SIS trilayer takes place, it is adaptable to a wide class of fabrication protocols and a wide variety of ferromagnetic materials.

**Figure 5:**
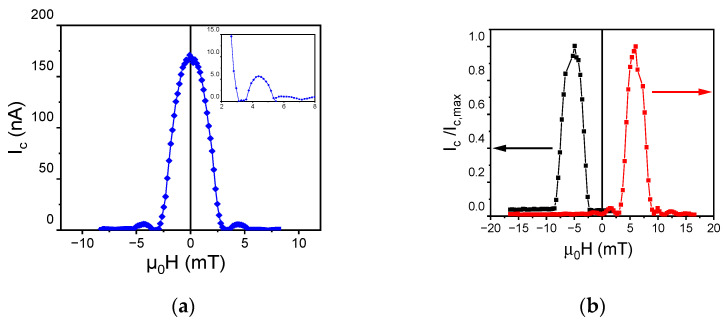
(**a**) Critical current vs. parallel magnetic field measured (*I_c_*(*H*) curve) measured at T = 10 mK for the SIS’FS junction with a diameter of 10 μm, showing the familiar Airy diffraction pattern. In the inset, a magnification of the two first secondary lobes is shown. (**b**) Hysteretic behavior of the *I_c_/I_c,max_* vs. *H* curve acquired applying a magnetic field larger than 30 mT. The black and the red curves are the magnetic pattern in the downward and upward direction of the magnetic field, respectively.

The high quality of the fabricated junctions is further proven by the dependence of the Josephson current on the external magnetic field, as shown in Figure 5a for a SIS’FS junction with a diameter of 10 μm. In the sweeping range (−10, 10) mT, irreversible magnetic processes are not activated [39]. Thus, the F layer does not acquire a remanent magnetization and a standard Airy pattern is observed (Figure 5a) [30]. The nodes in the *I_c_(H)* curves approaching *I_c_* = 0 indicate a uniform current distribution [30] and a lack of shorts in the surrounding oxide, and flux trapped throughout the junction electrodes [40]. 

By applying a field larger than 30 mT at *T* = 4 K (>*T_c_*), the F-layer acquires a remanent magnetization. Thus, after cooling down to *T* = 7 mK in zero field, the total magnetic flux Φ through the junction becomes $\Phi =\mu_{0}HDd_{m}+\mu_{0}M_{F}Dd_{F}$, where $M_{F}$ is the F magnetization, and $d_{m}=2\lambda_{L}+d_{s^{\prime }}+d_{F}+d_{ox}\;$ is the thickness of the material penetrated by the applied field H, with $d_{s},$ $d_{F}$, and $d_{ox}$ as the thicknesses of the intermediate Al ($d_{s^{\prime }}$ < $\lambda_{L}$), of the F, and oxide layer, respectively [17]. Since the maximum of *I_c_(H)* curves occurs at Φ=0, the *I_c_(H)* curves acquire a magnetic hysteresis due to the F-magnetization $M_{F}$ reversal [41,42] (Figure 5b). These measurements demonstrate that the SIS’FS junctions can be used as switchable magnetic elements.

## 4. Conclusions

In this paper, we report on the fabrication and characterization of high-quality micron-sized aluminum (Al) JJs by using an innovative fabrication process borrowed from niobium junction technology. It is based on optical lithography and the anodization of the Al top layer to define the geometry of the junction. An additional silicon dioxide insulating layer allows us to obtain very high-quality Al JJs down to 12 μm^2^. The fabrication process is extended to hybrid magnetic JJs (MJJs) based on a SIS’FS and from a technological point of view, is compatible with the implementation of most ferromagnetic materials. Moreover, it provides the capability to develop ad hoc switchable elements and guarantee easy integration of MJJs in a large variety of digital and quantum circuits, including transmon qubits, through a standard fabrication process since the F layer can be deposited ex situ without affecting the tunnel properties of the overall device. 

## Figures and Tables

**Table 1:** Parameter of Al-based junctions at T = 10 mK, where *D* is the diameter of the junction, *A* is the area, *J_c_* is the critical current density, and *R_n_* is the normal resistance at T = 10 mK. The energy gap at T = 0 K, Δ_0_, and the critical temperature *T_c_* are estimated as fitting parameters from the analysis of the *V_gap_* as a function of the temperature. *B* is a fitting parameter to consider the suppression of *I_C_R_N_* compared to the Ambegaokar-Baratoff expected value.

JJs	*D* (μm)	*J_c_* (A/cm^2^)	*R_n_* (kΩ)	*I_c_R_n_* (μV)	*R_n_A* (kΩμm^2^)	*2*Δ*_0_* (μV)	*T_c_* (K)	*B*
SIS’FS	4	0.34 ± 0.07	1.7	75	23	391 ± 2	1.28 ± 0.02	0.27 ± 0.04
SIS’FS	10	0.43 ±0.09	0.2	70	15	404 ± 3	1.30 ± 0.03	0.22 ± 0.05
SIS’S	4	0.34 ± 0.07	1.7	75	21	390 ± 2	1.30 ± 0.01	0.23 ± 0.01

## Data Availability

Not applicable.
